# Enterovirus 71 non-structural protein 3A hijacks vacuolar protein sorting 25 to boost exosome biogenesis to facilitate viral replication

**DOI:** 10.3389/fmicb.2022.1024899

**Published:** 2022-10-05

**Authors:** Zhihui Ruan, Yicong Liang, Zicong Chen, Jialing Yin, Chengcheng Li, Pan Pan, Qiwei Zhang, Jianguo Wu, Zhen Luo

**Affiliations:** ^1^Guangdong Provincial Key Laboratory of Virology, Institute of Medical Microbiology, Jinan University, Guangzhou, China; ^2^Foshan Institute of Medical Microbiology, Foshan, China

**Keywords:** enterovirus 71 (EV71), EV71 3A protein, vacuolar protein sorting 25, exosome, viral replication, hand, foot and mouth disease (HFMD)

## Abstract

Human enterovirus 71 (EV71) is one of the major agents of the hand, foot, and mouth disease (HFMD), and occasionally causes severe neurological complications. There is clinical evidence that EV71 infection increases the exosomes in the serum of severe HFMD patients, suggesting a role of exosomes in EV71 pathogenesis. However, the relationship between exosomes and EV71 replication remains elusive. In this study, we initially found that EV71 infection elevated exosome biogenesis in the cultured cells. Among EV71 non-structural proteins, we identified EV71 3A, but not 3B, constitutively promoted exosome secretion. In detail, EV71 3A protein interacted with vacuolar protein sorting 25 (VPS25), while knock-down of VPS25 reduced EV71 3A protein- and EV71-induced exosome production. Further studies revealed VPS25 located on exosomes and its expression correlated to the exosome production. During EV71 infection, knock-down of VPS25 decreased exosome biogenesis to attenuate viral replication. Consistently, GW4869, an exosome inhibitor, exerted an obviously antiviral activity against EV71 replication companied with the decrease of exosome secretion or formation. These findings suggest the binding of EV71 3A and VPS25 benefited exosome biogenesis, thereby boosting viral replication. This study uncovers a novel mechanism underlying EV71-mediated exosomes in the regulation of viral replication, which provides potential anti-viral strategies against the EV71 infection and transmission in HFMD.

## Introduction

Human enterovirus 71 (EV71) is one of the major pathogens responsible for hand, foot, and mouth disease (HFMD) with severe neurological complications (Solomon et al., 2010). EV71 belongs to the enterovirus genus in the *Picornaviridae* family, containing a single-stranded, positive-sense RNA of approximately 7.4 kb divided into a single large open reading frame (ORF), 5′- and 3′-untranslated regions (UTRs). After EV71 enters host cells, viral RNA is translated into a large polyprotein, which is segmented into four structural viral capsid proteins (VP4, VP2, VP3, and VP1) and seven non-structural proteins (2A, 2B, 2C, 3A, 3B, 3C, and 3D) (Wong et al., 2010). In the process of the viral life cycle, the endocytic pathway is important for enterovirus replication. Exactly, enterovirus non-structural proteins tether host lipid droplets and reorganize the secretory pathway to generate distinct organelles for RNA replication (Hsu et al., 2010; Laufman et al., 2019), which arranges cell membrane and associated proteins to generate extracellular vesicles.

The biogenesis pathway of extracellular vesicles has obvious overlapping processes with virus assembly and budding (Ju et al., 2021). Exosomes are a type of extracellular vesicles with a saucer-shape of 30–200 nm in diameter and released outside the cell in a form secreted after fusion of intracellular multivesicular bodies (MVBs) with the cell membrane (Thery et al., 2002; Kalluri and LeBleu, 2020). The microenvironment of cells may affect the content of exosomes and their biomarkers. Exosomes usually contain proteins from extracellular matrix and cell membrane, cytosol and nucleus, metabolites, and nucleic acids (Kalluri and LeBleu, 2020). The common proteins exist in almost all exosomes, for example, tetraspanins (such as CD9, CD63, and CD81), hepatocyte growth factor receptor tyrosine kinase substrate (HRS), and tumor susceptibility gene 101 (Tsg101) and thus are used as markers for exosome identification (Shao et al., 2018). In the immune system, exosomes are involved in immune cell interactions, including antigen presentation, immune-activating functions, immunosuppressive properties, and immune tolerance (Thery et al., 2009; Robbins and Morelli, 2014). The function of exosomes in viral infection and replication has been gradually uncovered. A dual role of exosomes is observed in the forms of both promotion and inhibition of viral infection (Alenquer and Amorim, 2015; Schorey et al., 2015). Nonetheless, accumulating evidence suggests that virus-modified exosomes contribute to viral infection and immune escape. The number of secreted exosomes and their content vary depending on their biogenesis, cellular origin, and cellular state. Clinically, the difference in exosomal microRNA content is highly relevant to the severity of HFMD caused by EV71 infection (Jia et al., 2014; Min et al., 2018). Current evidence suggests that exosomes can promote EV71 transmission in human neural (Mao et al., 2016; Too et al., 2016) and intestinal epithelial cells (Huang et al., 2020). It is also reported that exosomes promote EV71 infection by transferring miR-146a to suppress type I interferon responses (Fu et al., 2017), and cloak the virion to non-lytically transmit EV71 (Gu et al., 2020). However, the mechanism by which EV71 modulates exosome biogenesis remains unknown.

The role of the endosomal sorting complex required for transport (ESCRT) machinery in the multivesicular endosomes (MVEs) as intraluminal vesicles (ILVs) biosynthesis regulates exosome formation (van Niel et al., 2018). Through manipulating ESCRT components, several ESCRT subunits could act selectively on MVBs for exosome secretion (Vietri et al., 2020). ESCRT-II is required for the formation of MVBs and the sorting of endosomal cargo proteins into MVBs, and the ESCRT-II complex may be involved in the recruitment of the ESCRT-III complex (Vietri et al., 2020), which may be involved in promoting budding of certain RNA viruses (Pincetic et al., 2008). Vacuolar protein sorting 25 (VPS25) is a component of the ESCRT-II complex, and the protein subunits in the ESCRT-II complex are arranged in the form of the letter “Y” with VPS22 and VPS36 (Hierro et al., 2004; Wernimont and Weissenhorn, 2004). Thus, the potential function of VPS25 in the regulation of exosome biogenesis upon EV71 infection is of high interest.

In this study, we observed the difference in the cellular secretion of exosome upon EV71 infection, showing that EV71 infection and EV71 3A protein overexpression can promote the secretion of exosome. Through a protein-protein interaction screening, we found that EV71 3A protein physically binds to a component of ESCRT-II complex VPS25 protein, thereby promoting exosome secretion. Further studies demonstrated that the VPS25-mediated exosome biogenesis facilitates EV71 replication. Altogether, our findings highlighted a novel mechanism by which EV71 3A hijacks VPS25 to favor the biogenesis of exosome leading to the facilitation of viral replication.

## Materials and methods

### Cell culture

Human rhabdomyosarcoma cells (RD), embryonic 293T cells (HEK293T) were obtained from ATCC (Manassas, VA, USA). The cells were cultured in DMEM (Gibco, Carlsbad, CA, USA) containing 10% fetal bovine serum (FBS) (Gibco) or Exosome-depleted FBS (VivaCell Biosciences Inc., Shanghai, China), supplemented with 100 U/ml penicillin and 100 mg/ml streptomycin sulfate (Gibco) at 37°C in a 5% CO_2_ incubator.

### Virus infection

Human EV71 (Xiangyang-Hubei-09) was preserved in our laboratory (GenBank accession number JN230523.1). EV71 was propagated in RD cells. EV71 infected RD cells with different multiplicities of infection (MOIs) and the unbound virus was washed away after 2 h. Then, the infected cells were maintained with a fresh medium at 37°C. The determination of virus titer was performed by serial dilutions for the infectivity in RD cells detected by TCID50 (50% Tissue Culture Infective Dose) as previously described (Luo et al., 2014).

### Plasmid construction

The full-length *VPS25* gene (GenBank accession no. NM_032353.4) was cloned into pcDNA3.1-3 × FLAG vector to generate plasmid encoding FLAG-VPS25, and cloned into pBiFc-VN173 vector (Addgene plasmid #22010) to express VPS25 fusion protein using ClonExpress MultiS One Step Cloning Kit (Vazyme Biotech, Nanjing, China) as previously described (Luo et al., 2017). The DNA fragment of EV71 3A gene was ligated into eGFP-C1 vector to generate plasmids expressing GFP-tagged 3A and cloned into pBiFC-VC155 vector (Addgene plasmid #22011) to express 3A fusion protein, respectively. The information on primers is listed in Table 1.

**TABLE 1:** List of primers used in this study.

Primer title	Orientation (5′−3′)
FLAG-VPS25 F	AGTCCAGTGTGGTGGAATTCCATGGCGATGAGTTT CGAGTGGCCGTG
FLAG-VPS25 R	GCCCTCTAGACTCGAGCGGCCGCCTAGAAGAACTT GACGCCTCGGCC
GFP-3A F	TCGAGCTCGGCCCACCCAAGTTCAGGC
GFP-3A R	TCGAAGCTTTTGAAACCCTGCAAAGAGCTTGT
VN173-VPS25 F	AAAGACGATGACGACAAGCTTATGGCGATGAGTTT CGAGTG
VN173-VPS25 R	GATGGATCTTCTAGAGTCGACGAAGAACTTGACGC CTCGGCC
VC155-3A F	TGGCCATGGAG GCCCGAATTCACGGCCCACCC AAGT
VC155-3A R	TTTGCACGCCGGACGGGTACCTTGAAACCCTGCAA AGAGCTTGT

F, forward; R, reverse.

### Reagents

The specific small interfering RNAs (siRNA) to the negative control (NC) and *VPS25* gene were synthesized by Guangzhou RiboBio Co., Ltd. (Guangzhou, China). The sequences of siRNA were as followed: si-VPS25-1#: 5′-GCA CAAGGCCGAGATCATC; si-VPS25-2#: 5′-GGGAAACTCA TCTATCAGT; si-NC: 5′-TTCTCCGAACGTGTCACGT. An exosome inhibitor GW4869 (Catalog number: HY-19363) and membrane dyes Dil (Catalog number: HY-D0083) were purchased from MedChemExpress (MCE) Corporation (Shanghai, China). The Cell Counting Kit 8 (CCK8) was purchased from Dojindo Laboratories (Kumamoto, Japan). 4′,6-Diamidine-2′-phenylindole dihydrochloride (DAPI) (Catalog number: 28718-90-3) was purchased from Sigma-Aldrich (St. Louis, MO, USA).

### Antibodies

CoraLite^®^488-conjugated CD9 monoclonal antibody (Catalog number: CL488-60232), HRP-conjugated GFP monoclonal antibody (Catalog number: HRP-66002), rabbit IgG control polyclonal antibody (Catalog number: 30000-0-AP), mouse antibody against β-actin (Catalog number: 66009-1-Ig), and rabbit antibodies against VPS25 (Catalog number: 15669-1-AP), GFP (Catalog number: 50430-2-AP), HRS (Catalog number: 10390-1-AP), and Tsg101 (Catalog number: 28283-1-AP) were purchased from ProteinTech Group (Wuhan, China). Mouse antibody against CD63 (Catalog number: sc-5275) was purchased from Santa Cruz Biotechnology (Santa Cruz, CA, USA). Rabbit antibodies against CD9 (Catalog number: 98327S) and calnexin (Catalog number: 2679) were purchased from Cell Signaling Technology (Beverly, MA, USA). Rabbit antibody against HA (Catalog number: H6908) and mouse antibody against FLAG (Catalog number: F3165) were purchased from Sigma-Aldrich (St. Louis, MO, USA). Rabbit antibody against EV71 VP1 (Catalog number: GTX132339) was purchased from GeneTex, Inc. (Irvine, CA, USA). Rabbit antibody against EV71 3C (Catalog number: A10003) was purchased from ABclonal Technology (Wuhan, China). Mouse antibody against EV71 3A was kindly provided by Dr. Yongbo Yang of Central China Normal University.

### Exosome isolation and purification

Cells were cultured in a medium with Exosome-depleted FBS for 24 h. Exosome isolation and purification was subjected to the warranted performance as previously described (Thery et al., 2006; Lobb et al., 2015). Firstly, the cell supernatant was collected and centrifuged at 2,000 × *g* for 10 min and 10,000 × *g* for 30 min to remove dead cells and debris, respectively. Then, the exosomes were then isolated by using an ultra-centrifuge (Optima XE-100, Beckman Coulter, Indianapolis, IN, USA) at 100,000 × *g* at 4°C for 70 min. The harvested exosomes were washed in PBS and subjected to the repeated ultracentrifugation as above to remove contaminating proteins. Finally, the purified exosomes were resuspended in PBS and stored in ultra-low adhesion microcentrifuge tubes at −80°C.

The quantification of exosome preparations is based on the same volume of cell supernatants from different groups. To compare the exosome production, the equal volume of exosomes in PBS of each sample with a total protein amount ranging from 0.5 to 2.0 μg was loaded in gels for Western blot analysis. After cell supernatants were collected, the cells in the plated were digested with trypsin and further washed with PBS. The suspended cell was subjected to centrifugation at 4,000 × *g* for 5 min. The sedimented cells were lysed in RIPA buffer to prepare cell lysis for Western blot analysis.

### Transmission electron microscopy and immunoelectron microscopy

Briefly, exosome samples were adsorbed at activated formvar/carbon-coated grids, fixed in 2.5% glutaraldehyde for 30 min, pelleted by ultracentrifugation, and placed on grids, followed by negative staining using 1% uranyl acetate in water. Grids were examined using a JEM1400 120 kV Transmission Electron Microscope (JEOL Ltd., Peabody, MA, USA). Immunogold labeling was performed as previously described (Peters et al., 2021). For immunogold labeling, exosome samples were added and grids were plated with rabbit polyclonal antibody against VPS25 or isotype IgG at a ratio of 1:50 dilution in blocking buffer (0.5% BSA, 0.5% ovalbumin in PBS) for incubation for 1 h. Followed by triple washes for 5 min in PBST, the 10 nm gold-labeled goat anti-rabbit secondary antibody at 1:50 dilution was used to incubate samples for 1 h incubation. After three additional washes in PBST, samples were fixed in 8% glutaraldehyde for 30 s. Images were captured using the Transmission Electron Microscope.

### Nanoparticle tracking analysis

The exosome samples were prepared in an optimal dilution. The size and count of exosomes were assessed by nanoparticle tracking analysis (NTA) (NTA 3.4 Build 3.4.003) on NanoSight NS300 (Malvern Panalytical, Malvern, UK).

### Immunoprecipitation and immunoblot analysis

HEK293T cells were cultured in the 6-cm dish and harvested after transfection with plasmids for 48 h. The cells were lysed in 800 μl RIPA buffer and 80 μl lysate was reserved for direct immunoblot analysis. The rest of lysate was incubated with primary antibody overnight together with Protein G Agarose (GE Healthcare, Milwaukee, WI, USA) for another 2 h. After five rounds of washes, proteins were fractionated by SDS-PAGE and transferred to nitrocellulose membrane. Non-specific sites were blocked with 5% skim milk for 1 h at room temperature. After three times of PBST wash, the nitrocellulose membrane was incubated with primary and secondary antibodies. Blots were analyzed using a ChemiDoc imaging system (Bio-Rad Laboratories, Hercules, CA, USA).

### Immunofluorescence microscopy

Rhabdomyosarcoma cells were seeded on 20-mm coverslips and infected with EV71. Then, the medium was removed and cells were washed with PBS, fixed with 4% formaldehyde for 30 min, and permeabilized with 0.2% Triton X-100 for 10 min at room temperature. After another PBS wash, cells were blocked in PBS containing 5% BSA for 1 h and incubated with mouse anti-EV71-3A antibody dilution (v/v = 1:200) and rabbit anti-VPS25 antibody dilution (v/v = 1:200) overnight at 4°C. Samples were incubated with FITC-conjugated goat anti-mouse and Cy3-conjugated goat anti-rabbit IgG dilution (v/v = 1:500) (ProteinTech Group, Wuhan, China) for 45 min at room temperature. For nuclei staining, 1 μg/ml DAPI in methanol was used and incubated with samples for 10 min at room temperature. After washing with PBS, the cells were observed by Leica TCS SP8 confocal laser scanning microscopy (Leica Microsystem, Wetzlar, Germany).

For the immunofluorescence staining of exosomes, exosomes were labeled with Dil membrane dye as previously described (Jiang Y. et al., 2020) and CoraLite^®^488-conjugated CD9 antibody dilution (v/v = 1:200). Labeled exosomes were washed with PBS and ultracentrifuged at 100,000 × *g* for 70 min at 4°C for three times. The samples were dropped on a glass slide and observed under Leica TCS SP8 confocal laser scanning microscopy with Lightning mode.

### Bimolecular fluorescence complementation assay

The bimolecular fluorescence complementation (BiFc) assay was performed to examine physical protein-protein interaction as previously described (Luo et al., 2017; Pan et al., 2021). The plasmids expressing VN173, VC155, or fusion protein VN173-VPS25, VC155-3A were co-transfected into HEK293T cells using Lipofectamine 2000 (Invitrogen, Carlsbad, CA, USA). At 24 h post-transfection, cells were pre-cultured at 4°C for 10 min. The fusion proteins in living cells were observed by Leica TCS SP8 confocal laser scanning microscopy.

### Immunocapture-based enzyme-linked immunosorbent assay of exosome

The enzyme-linked immunosorbent assay (ELISA) measurement of protein on the surface of exosome was performed as previously reported (Chen et al., 2018). For the detection of VPS25 on exosome, ELISA plates (96-well) (Nunc Cell Culture) were coated with 0.1 μg/per well (100 μl) of mouse anti-CD63 antibody overnight at 4°C. Free binding sites were blocked with 200 μl of 5% BSA for 1 h at room temperature. Then, 100 μl of exosome samples purified from the equal volume of cell culture supernatants suspended in PBS, were added to each well. Rabbit anti-VPS25 antibody dilution (v/v = 1:200) was added to each well and incubated for 1 h at room temperature. HRP conjugated secondary antibody (Catalog number: SA00001-2) (ProteinTech Group, Wuhan, China) diluted in PBS containing 0.1% BSA was incubated for 1 h at room temperature. Plates were developed with TMB Substrate Solution (Catalog number: P0209-100 ml) (Beyotime Biotechnology, Jiangsu, China) and stopped with H_2_SO_4_. The OD values in plates were determined at 450 nm with a Varioskan LUX multimode microplate reader (Thermo Scientific, Waltham, MA, USA).

### Statistics

Statistical analysis was performed by Student’s *t*-test using Prism 7 software (GraphPad Software Inc., San Diego, CA, USA). A *P*-value < 0.05 was considered to indicate statistical significance.

## Results

### Enterovirus 71 infection promotes exosome biogenesis

**FIGURE 1:**
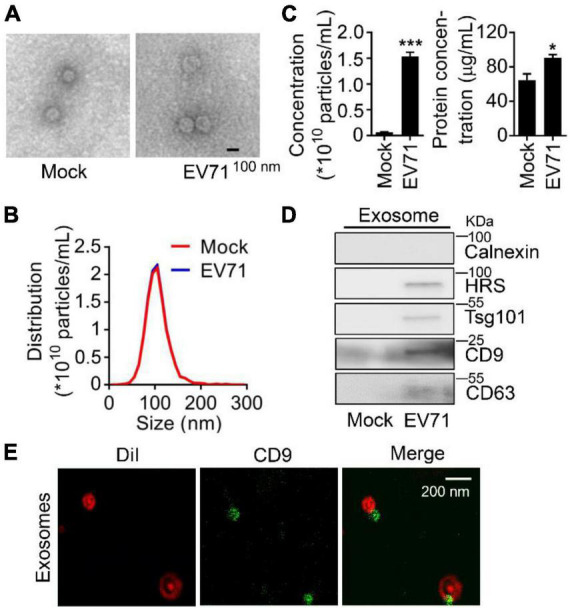
Enterovirus 71 (EV71) infection promotes exosome biogenesis. RD cells were mock-infected or infected with EV71 (MOI = 0.5). At 24 h post-infection, cell supernatants were harvested for exosome extraction. **(A–C)** The characteristics of exosomes were examined by transmission electron microscope (TEM) and nanoparticle tracking analysis (NTA) to observe the morphology **(A)**, size and distribution **(B)**, particles and protein concentration **(C)** of exosomes, respectively. The size is expressed by diameter. **(D)** The extracted exosomes were detected by Western blot analysis. HRS, Tsg101, CD9, and CD63 are used as exosome markers, and Calnexin is a marker of the cytosol to reflect cellular contamination. **(E)** The extracted EV71-induced exosomes were stained by Dil (red) and fluorophore-conjugated CD9 antibody (green), then examined by Leica TCS SP8 confocal laser scanning microscopy with Lightning mode. WCL, whole cell lysate. Graph shows as mean ± SD. *P < 0.05; ***P < 0.001.

To investigate the relationship between EV71 infection and exosome secretion, we preliminarily determined whether EV71 infection influenced the secretion of cellular exosomes. In both mock and EV71-infected cell supernatants, the extracted extracellular vesicles were observed with a typical exosome structure by transmission electron microscopy (TEM) assay (Figure 1A) and measured with a diameter of around 104 nm by NTA (Figure 1B). Subsequently, the particle and protein concentration of extracted exosomes in the samples was counted. It was displayed that both particle number and protein concentration of exosome secreted from EV71-infected cells was significantly increased than that of uninfected ones (Figure 1C). The content of exosomal markers, HRS, Tsg101, CD9, and CD63 proteins in the extracted exosomes (Fernandez-Llama et al., 2010; Jiang Z. et al., 2020) were further verified by Western blot assay. The purification of extracted exosome was confirmed by the absence of Calnexin protein (Figure 1C, upper panel). Meanwhile, the protein levels of HRS, Tsg101, CD9, and CD63 were elevated in the exosome secretion by EV71 infection (Figure 1D). To further confirm the identity of the vesicles recovered from the cultured cells supernatant, we performed immunostaining using membrane dye Dil and fluorophore-conjugated CD9 antibody on the exosome preparation. It was presented that the exosomes were specifically stained with Dil companied with colocalization with CD9 protein (Figure 1E). Altogether, these data indicated that EV71 infection promotes the secretion of exosome in host cells.

### Enterovirus 71 non-structural protein 3A participates in the increased exosome biogenesis

**FIGURE 2:**
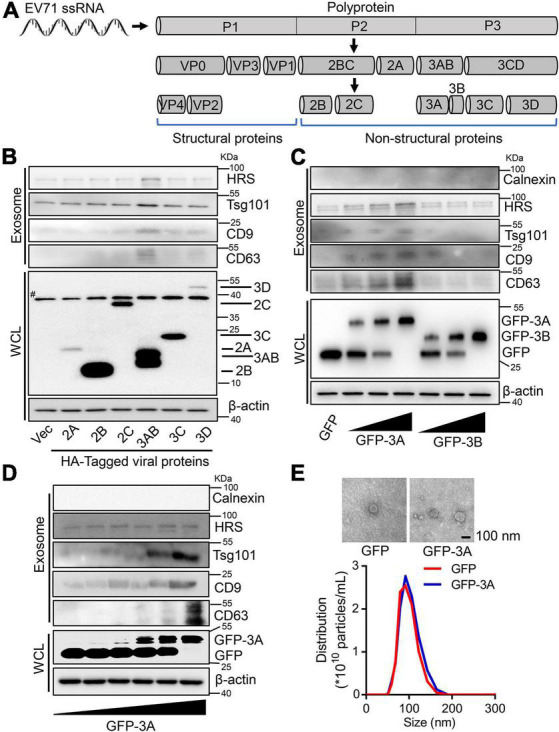
Enterovirus 71 (EV71) 3A protein increases exosome biogenesis. **(A)** Schematic diagram of EV71 structure and non-structural proteins. **(B–D)** RD cells were transfected with 2 μg plasmids expressing HA-tagged EV71 non-structural proteins **(B)**, or different doses (0, 0.5, 1, and 2 μg) of plasmids expressing GFP-tagged 3A or 3B protein **(C)**, or different doses (0, 0.2, 0.5, 1, 1.5, and 2 μg) of plasmids expressing GFP-tagged 3A protein **(D)**. At 24 h post-transfection, the extracted exosomes from cell supernatants and cell lysates were collected for Western blot analysis. HRS, Tsg101, CD9, and CD63 are used as exosome markers, while Calnexin is a marker of cytosol. β-actin is presented as an internal reference protein in cell lysates. WCL, whole cell lysate. #, the non-specific band. **(E)** RD cells were transfected with 2 μg plasmids expressing GFP or GFP-tagged 3A protein. At 24 h post-transfection, the extracted exosomes from cell supernatants were observed by TEM and measured by size and distribution, respectively.

Considering that enterovirus non-structural proteins reorganize host lipid droplets and secretory pathways to arrange cell membrane and cargo proteins to generate extracellular vesicles (Hsu et al., 2010; Laufman et al., 2019), we further explore the regulation of EV71 non-structural proteins on secretion of exosomes. Initially, we constructed and expressed a series of EV71 non-structural proteins (Figure 2A). Among six non-structural proteins, EV71 3AB protein obviously increased HRS, Tsg101, CD9, and CD63 proteins levels in the collected exosomes (Figure 2B), suggesting EV71 3AB protein could stimulate the production of exosome. Then, the increased expression of 3A protein significantly promoted HRS, Tsg101, CD9, and CD63 protein levels in the exosomes (Figure 2C, left panel), whereas the increased expression of 3B protein failed to affect the levels of corresponding proteins in the exosomes (Figure 2C, right panel), indicating EV71 3A, but not 3B protein constructively enhanced exosome secretion. Consistently, overexpression of GFP-3A protein constitutively enhanced HRS, Tsg101, and CD9 protein levels in the exosomes in a dose-dependent manner (Figure 2D). Of note, the extracted exosomes from the supernatants of GFP and GFP-3A expressed cells were detected by TEM observation (Figure 2E, upper panel). Consistently, NTA data revealed that the diameter of purified exosomes was not affected by either overexpression of GFP or GFP-3A protein (Figure 2E, lower panel), while both particles and protein concentration of exosomes was increased by GFP-3A but not GFP protein, suggesting an increase of exosome secretion (Supplementary Figure 1). Therefore, these results concluded that EV71 3A protein specifically increases exosome biogenesis.

### Enterovirus 71 3A protein interacts with vacuolar protein sorting 25

**FIGURE 3:**
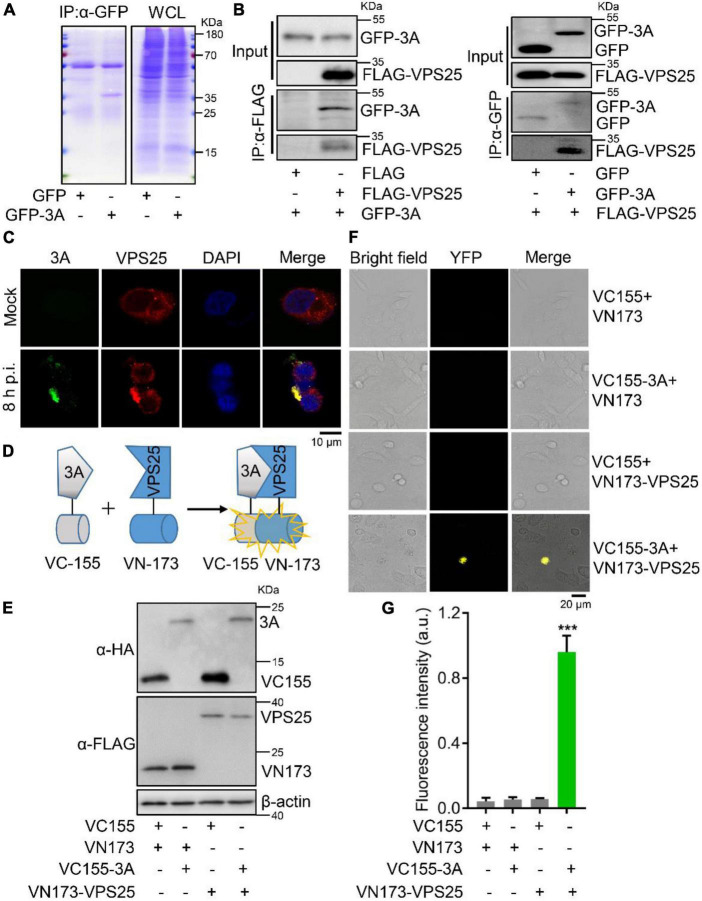
Enterovirus 71 (EV71) 3A protein interacts with VPS25. **(A)** RD cells were transfected with plasmids expressing GFP or GFP-tagged 3A. Cell lysates were immunoprecipitated with anti-GFP (α-GFP) antibody. The protein samples were separated by SDS-PAGE and visualized with Coomassie blue staining. **(B)** HEK293T cells were co-transfected with empty or indicated plasmids expressing FLAG-tagged VPS25 or GFP-tagged 3A, followed by the immunoprecipitated with anti-FLAG (α-FLAG) or anti-GFP (α-GFP) antibody, respectively. The corresponding proteins were detected by Western blot analysis. **(C)** RD cells were infected with EV71 (MOI = 0.5) for 8 h. Immunofluorescence assay was performed after labeling proteins of EV71 3A (green), VPS25 (red), and staining nuclei with DAPI (blue). **(D)** Schematic of bimolecular fluorescence complementation assay (BiFc). **(E–G)** RD cells were co-transfected with Venus BiFc plasmids expressing control protein (VC155 or VN173), 3A protein (VC155-3A), and VPS25 protein (VN173-VPS25), respectively. At 24 h post-transfection, the expression of corresponding proteins was detected by Western blot analysis **(E)** or visualized by confocal microscopy **(F)**. The fluorescence intensity was analyzed using ImageJ software **(G)**. YFP, yellow fluorescence protein; a.u., arbitrary unit. Graph shows as mean ± SD. ***P < 0.001.

Since specialized ESCRT components could act on MVBs for exosome secretion (Vietri et al., 2020), the mechanism underlying EV71 3A protein promoting the secretion of exosome was further investigated. In the expressed GFP or GFP-3A protein cell lysates, we performed a co-immunoprecipitation (Co-IP) assay using anti-GFP antibody to separate the possible cellular proteins interacting with 3A protein by SDS-PAGE (Figure 3A), and the precipitated protein samples were subjected to mass spectrometry analysis (Table 2). As a typical component of the ESCRT-II complex (Wernimont and Weissenhorn, 2004), VPS25 potentially interacted with EV71 3A protein. Next, Co-IP assays revealed that VPS25 could immunoprecipitate 3A protein using anti-FLAG antibody (Figure 3B, left), or 3A protein immunoprecipitated VPS25 using anti-HA antibody (Figure 3B, right).

**TABLE 2:** List of potential proteins interacting with EV71 3A protein.

Protein name	Description or alias
Vimentin	HUMAN Vimentin
VPS25	HUMAN Vacuolar protein sorting 25
hnRNPA2B1	HUMAN Heterogeneous nuclear ribonucleoprotein A2/B1
hnRNPA3	HUMAN Heterogeneous nuclear ribonucleoprotein A3
hnRNPA1	HUMAN Heterogeneous nuclear ribonucleoprotein A1
Nucleolin	HUMAN Nucleolin
ACTG	HUMAN Actin, cytoplasmic 2
HSP90AB1	HUMAN Heat shock protein HSP 90-beta
hnRNPA/B	HUMAN Heterogeneous nuclear ribonucleoprotein A/B
GRP75/HSPA9	HUMAN Stress-70 protein, mitochondrial
HSP60	HUMAN 60 kDa heat shock protein, mitochondrial
hnRNPU	HUMAN Heterogeneous nuclear ribonucleoprotein U
HSP90B1	HUMAN Endoplasmin
HSPB1	HUMAN Heat shock protein beta-1
HSP72	HUMAN Heat shock-related 70 kDa protein 2
hnRNPC1/C2	HUMAN Heterogeneous nuclear ribonucleoproteins C1/C2

To confirm this interaction, RD cells were infected with EV71 at 8 h p.i. Notably, we observed that VPS25 protein colocalized with EV71 3A protein in the cytosol (Figure 3C), indicating the occurrence of robust interaction between 3A and VPS25 proteins during viral replication (Supplementary Figure 2). To further verify the physical interaction of EV71 3A and VPS25, the BiFc assay was employed in the living cells (Figure 3D). The indicated fusion protein with the complementary yellow fluorescence protein (YFP) fragment VC155-3A and VN173-VPS25 were expressed (Figure 3E). In the absence of either VC155-3A or VN173-VPS25, the YFP in cells failed to be observed as expected (Figure 3F), while in the presence of VC155-3A and VN173-VPS25, the YFP in cells was observed (Figure 3F) and intensity of fluorescence was robustly detected (Figure 3G), suggesting a strong physical interaction between 3A and VPS25 proteins. Taken together, these results illustrated that EV71 3A physically interacts with VPS25.

### Vacuolar protein sorting 25 locates on exosome during Enterovirus 71-induced exosome biogenesis

**FIGURE 4:**
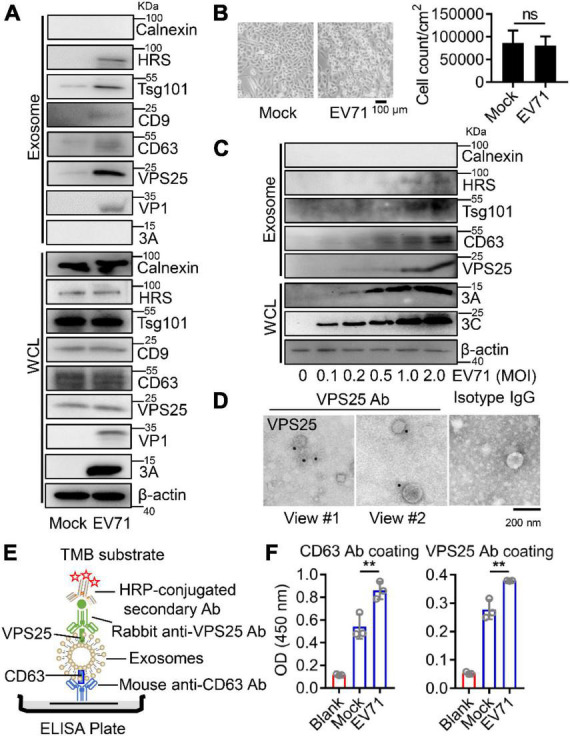
The location of VPS25 in EV71-induced exosome biogenesis. **(A–C)** RD cells were mock-infected or infected with EV71 (MOI = 0.5) for 24 h **(A,B)**. The cell images and count were captured **(B)**. RD cells were infected with EV71 at different MOIs **(C)**. At 24 h post-infection, extracted exosomes and cell lysates were analyzed by Western blot. **(D)** The extracted exosomes from panel **(A)** were detected by immunoelectron microscopy using anti-VPS25 antibody. **(E)** Schematic of immunocapture-based ELISA to detect protein on the surface of exosomes. **(F)** RD cells were mock-infected or infected with EV71 (MOI = 0.5). At 24 h post-infection, exosomes extracted from 10 mL of cell supernatant were detected by ELISA in the CD63 and VPS25 antibodies coating plates, respectively. Ab, antibody. Graph shows as mean ± SD. ns, not-significant. **P < 0.01.

Because of the interaction between VPS25 and EV71 3A, we next examined the impact of VPS25 on EV71 3A-induced exosome secretion. We found the protein levels of HRS, Tsg101, CD9, and CD63 along with VPS25 were elevated in the exosome secretion upon EV71 infection (Figure 4A, upper panel), whereas the expression of the above proteins was not significantly increased in whole-cell lysates during EV71 infection (Figure 4A, lower panel). Interestingly, VP1 but not 3A protein could be observed in EV71-induced exosomes (Figure 4A, upper panel), suggesting the evidence of EV71 virion in exosome as previously reported (Gu et al., 2020). We also observed that the number of cells was not significantly changed after EV71 infection (Figure 4B). In addition, the protein levels of HRS, Tsg101, and CD63 along with VPS25 increased in a dose manner upon EV71 infection (Figure 4C), suggesting a positive correlation between VPS25 protein level and the generation of exosomes.

We suspected that VPS25 protein could locate on the exosomes. In an immunoelectron microscopy experiment, VPS25 protein was specifically labeled and visualized on exosomes compared to a control (Figure 4D). To further verify this specific location, an ELISA measurement of VPS25 protein on the surface of exosome was introduced as previously reported (Chen et al., 2018). In this assay, the purified exosomes from cell culture supernatants were initially captured by the anti-CD63 antibody coating in a plate, and then detected exosomal VPS25 protein with anti-VPS25 antibody (Figure 4E). Using the immunocapture-based ELISA, exosomal VPS25 protein was specifically detected and increased in EV71-induced exosome captured by the anti-CD63 antibody compared to mock control (Figure 4F, left panel). In turn, exosomal CD63 protein was also detected with a higher level in EV71-induced exosome captured by the anti-VPS25 antibody compared to mock control (Figure 4F, right panel). Thus, we discovered that VPS25 protein correlates to the production of exosomes and locates on exosomes.

### Vacuolar protein sorting 25 is responsible for enterovirus 71-induced exosome biogenesis

**FIGURE 5:**
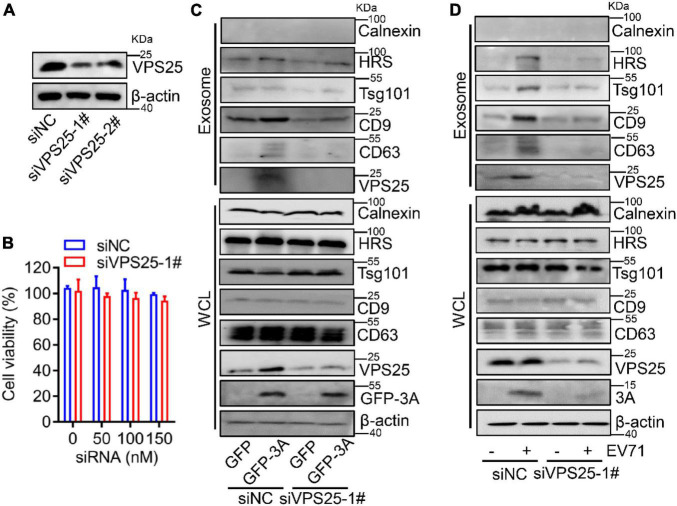
Vacuolar protein sorting 25 (VPS25) mediates EV71-induced exosome biogenesis. **(A,B)** RD cells were transfected with 100 nM siRNA negative control (siNC), or against VPS25 (siVPS25-1#, -2#), respectively. At 48 h post-transfection, the level of VPS25 protein was measured by Western blot **(A)**. Until 72 h post-transfection, the cell viability was measured using the CCK8 assay **(B)**. **(C,D)** RD cells were transfected with siNC or siVPS25-1# for 24 h and then transfected with plasmids expressing GFP or GFP-tagged 3A **(C)**, or infected with EV71 (MOI = 0.5) **(D)** for another 24 h. The extracted exosomes and cell lysates were analyzed by Western blot. The equal volume of exosomes in PBS of each sample with a total protein amount ranging from 0.5 to 2.0 μg was loaded. WCL, whole cell lysate.

To confirm the association between VPS25 protein and exosome generation, we utilized siRNA to silence endogenous VPS25 protein expression. The knock-down efficiency of VPS25 was accessed and the protein level of VPS25 was robustly decreased by siVPS25-1# relative to siVPS25-2# or siNC (Figure 5A). In addition, there was no significant cytotoxicity when cells were transfected with either siNC or siVPS25-1# at the concentration ranging from 50 to 150 nM (Figure 5B). Subsequently, we reduced the expression of VPS25 protein by using siRNA and then transfected plasmids expressing GFP or GFP-3A to cells. The knock-down of VPS25 did not affect the endogenous expression of Calnexin, HRS, Tsg101, CD9, and CD63 in cell lysates (Figure 5C, lower panel). The exosome secretion was enhanced in the presence of 3A protein, whereas this induction was restored by siVPS25-1# (Figure 5C, upper panel), demonstrating that VPS25 mediates EV71 3A-induced exosome biogenesis.

Because of the role of VPS25 in 3A-promoted exosome secretion, we explored the relationship between VPS25 protein and exosome upon EV71 infection. The cells were transfected with siVPS25-1# or siNC and then infected with EV71. We observed that the knock-down of VPS25 did not affect the endogenous expression of Calnexin, HRS, Tsg101, CD9, and CD63 in cell lysates (Figure 5D, lower panel). The exosome secretion was induced by EV71 infection, whereas this induction was restored by siVPS25-1# (Figure 5D, upper panel), indicating VPS25 mediates EV71-induced exosome biogenesis. Notably, we found cellular EV71 3A expression was decreased in the presence of siVPS25-1# upon EV71 infection (Figure 5D, lower panel). Altogether, VPS25 is important for EV71-induced exosome biogenesis.

### Vacuolar protein sorting 25 mediates enterovirus 71-induced exosome to facilitate viral replication

**FIGURE 6:**
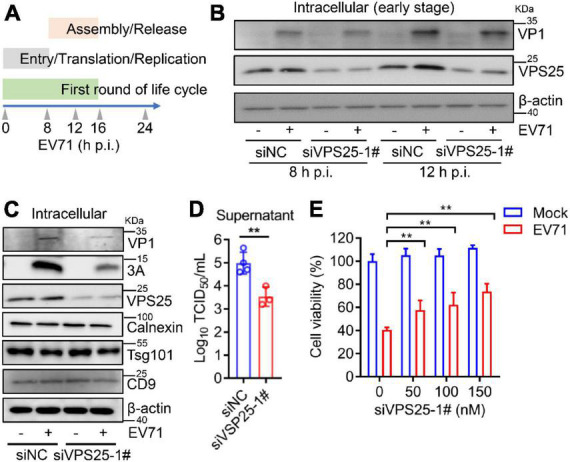
Vacuolar protein sorting 25 (VPS25) mediates EV71-induced exosome to facilitate viral replication. **(A)** Schematic of kinetic time points in the EV71 life cycle. **(B–D)** RD cells were transfected with 100 nM siNC or siVPS25-1# for 24 h and then infected with EV71 (MOI = 0.5). At 8 and 12 h p.i., the cell lysates were analyzed by Western blot **(B)**. At 24 h p.i., the cell lysates were analyzed by Western blot **(C)** and the progeny virus titer was measured by TCID50 assay **(D)**. **(E)** RD cells were transfected with different doses of siNC or siVPS25-1# (0, 50, 100, and 150 nM) for 24 h and then infected with EV71 (MOI = 0.5) for another 24 h. Cell viability was measured using the CCK8 assay. Graph shows as mean ± SD. **P < 0.01.

In the life cycle of enteroviruses, the first round of infection is deemed to be before 16 h post-infection (Su et al., 2020). In order to examine whether VPS25 affects EV71 intracellular replication, we designed kinetic time points within the first round of EV71 infection (Figure 6A). At both 8 and 12 h post-EV71 infection as an early stage, the viral protein VP1 and 3A expression was not affected in the cells transfected with siVPS25-1# relative to siNC (Figure 6B). Next, the function of VPS25-mediated exosome in EV71 replication was investigated. In the cells transfected with siVPS25-1# upon EV71 infection for 24 h, the viral expression of protein VP1 and 3A was significantly decreased (Figure 6C), suggesting inhibition of intracellular EV71 replication beyond the first round of infection. In a parallel experiment, the titer of progeny EV71 was significantly reduced by siVPS25-1# (Figure 6D). Moreover, the EV71-induced cytotoxicity was significantly reduced when cells were transfected with siVPS25-1# in a dose-dependent manner (Figure 6E). Altogether, the data demonstrated that VPS25 mediates EV71-induced exosome to benefit viral replication.

### GW4869 inhibits viral replication by the repression of enterovirus 71-induced exosome biogenesis

**FIGURE 7:**
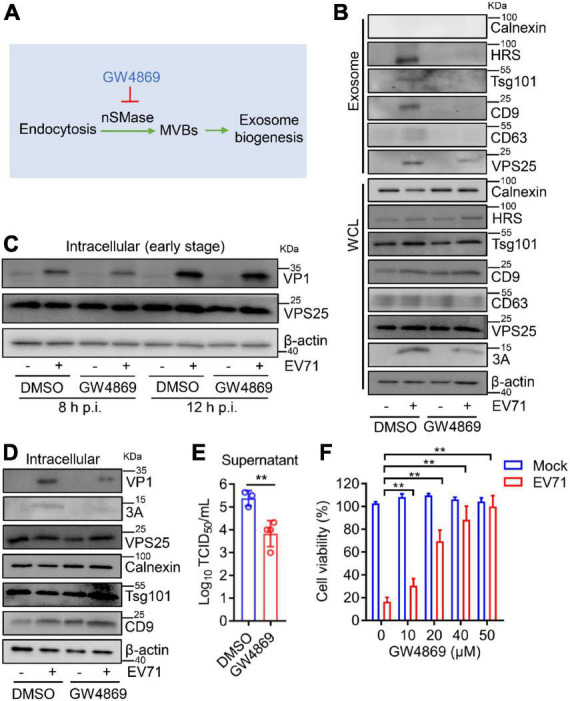
GW4869 inhibits viral replication via reducing exosome biogenesis. **(A)** GW4869 acts as an exosome inhibitor in the endocytic pathway. **(B–F)** RD cells were incubated with GW4869 (20 μM) for 2 h and then infected with EV71 (MOI = 0.5). At 24 h p.i., the extracted exosomes and cell lysates were analyzed by Western blot **(B)**. At 8 and 12 h p.i., the cell lysates were analyzed by Western blot **(C)**. At 24 h p.i., cell lysates were analyzed by Western blot **(D)** and progeny virus titer was measured by TCID50 assay **(E)**. WCL, whole cell lysate. **(F)** RD cells were incubated with different doses of DMSO or GW4869 (0, 10, 20, 40, and 50 μM) for 2 h and then infected with EV71 (MOI = 0.5) for another 24 h. Cell viability was measured using the CCK8 assay. Graph shows as mean ± SD. **P < 0.01.

Then, GW4869, an inhibitor of exosome biogenesis/release (Essandoh et al., 2015), which targets nSMase in an endocytic pathway to modulate exosome biogenesis (Figure 7A), was applied to test the effect of exosomes on EV71 replication. In cell lysates, GW4869 did not affect the endogenous expression of Calnexin, HRS, Tsg101, CD9, CD63, and VPS25 (Figure 7B, lower panel). In exosomes, the protein level of VPS25 companied with HRS, Tsg101, CD9, and CD63 was increased by EV71, whereas this induction significantly reduced in the presence of GW4869 (Figure 7B, upper panel). We also noticed that the cellular EV71 3A expression were obviously decreased in the presence of GW4869 upon EV71 infection (Figure 7B, lower panel), suggesting EV71-induced exosome biogenesis plays a role in viral replication.

**FIGURE 8:**
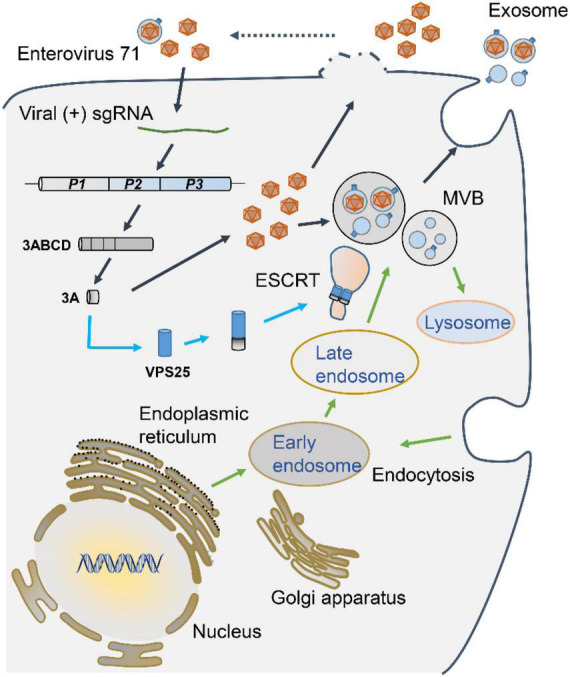
A proposed mechanism underlying VPS25 mediates EV71-induced exosome biogenesis to facilitate viral replication. Upon EV71 infection, viral single positive-sense RNA utilizes host translation machinery to translate a polyprotein, which is cleaved into structural and non-structural protein. Among viral non-structural proteins, EV71 3A protein physically interacts with VPS25, a component of the ESCRT-II complex, which participates in MVB generation and exosome biogenesis. The release of progeny EV71 could be in the forms of lytical and non-lytical manners. VPS25-mediated exosomes are engaged in the EV71 transmission and infection, thereby facilitating EV71 replication.

To further investigate the exosome in EV71 replication, the impact of GW4869 on EV71 intracellular replication was accessed. At both 8 and 12 h p.i., GW4869 did not affect the expression of EV71 VP1 protein (Figure 7C), implying that GW4869 had no obvious effect on EV71 intracellular replication at the early stage. At 24 h p.i., in the cells treated with GW4869 upon EV71 infection, the viral protein VP1 and 3A expression were significantly repressed (Figure 7D). Parallelly, the titer of progeny EV71 was obviously decreased by GW4869 (Figure 7E). Additionally, the EV71-induced cytotoxicity was significantly restored when cells were exposed to GW4869 in a dose-dependent manner (Figure 7F). These results revealed that EV71-induced exosome biogenesis plays a positive role in viral replication. Collectively, our study proposed a novel mechanism by which EV71 3A protein interacts with a component of the ESCRT-II complex, VPS25 to mediate exosome biogenesis and the release of progeny EV71, leading to facilitating viral infection and replication (Figure 8).

## Discussion

Upon viral infection, the modulation of the biosynthesis of exosome is involved in viral infection and etiology. For example, exosomes exploit interferon-alpha-induced intercellular antiviral activity against hepatitis B virus (HBV) (Yao et al., 2018). Exosomes mediate Zika virus (ZIKV) to support cell-to-cell transmission in cortical neurons (Zhou et al., 2019). Regarding EV71 infection, exosomes benefit the spread of virus and facilitate viral replication (Mao et al., 2016; Fu et al., 2017; Huang et al., 2020). Clinically, the changes in exosomes are highly relevant to the severity of HFMD caused by EV71 infection (Jia et al., 2014; Min et al., 2018). Here, we demonstrate that EV71 infection promotes the secretion of exosomes. However, the molecular mechanisms of the regulation on exosome generation during EV71 infection are not fully understood.

Based on the fact that enterovirus non-structural proteins reorganize host lipid droplets and secretory pathways to arrange cell membrane and cargo proteins to generate extracellular vesicles (Hsu et al., 2010; Laufman et al., 2019), we figured out that EV71 3A protein specifically induced exosome biogenesis. Further studies explored that EV71 3A physically interacted with VPS25, a component of the ESCRT-II complex, to mediate exosome biogenesis. The 3A protein of Picornavirus interacts directly or indirectly with many host cell proteins. It has been reported that the 3A proteins from multiple picornaviruses utilize the Golgi Adaptor Protein ACBD3 to recruit phosphatidylinositol 4-kinase class III beta (PI4KIIIβ) to promote virus replication (Greninger et al., 2012; Xiao et al., 2017). It also is reported that the 3A protein of enterovirus is a small hydrophobic protein and promotes viral replication by promoting the binding of capsule membrane to form replication complex (RC) and replication organelles (RO) (Arita et al., 2010; Ishikawa-Sasaki et al., 2014). In this study, we uncover a novel regulatory manner that EV71 3A constitutively promoted exosome secretion, which extends an exact function from 3A protein of EV71 on the regulation of exosome biogenesis.

Endosomal sorting complex required for transport complex is an ancient membrane remodeling and fragmentation system, mainly manipulating from MVBs to exosomes or extracellular vesicles biogenesis (Hurley, 2015; Guo et al., 2021). VPS25, belonging to the ESCRT-II complex members, plays an important role in the formation of exosomes (Juan and Furthauer, 2018). Our results provide direct evidence that VPS25 protein is present on exosomes and mediates both EV71- and EV71 3A-induced exosome biogenesis. It could be explained that the interaction of EV71 3A and VPS25 proteins participates in ESCRT pathway and acts on MVBs generation, thereby facilitating exosome biogenesis. However, the detailed events of the dynamic regulation of ESCRT complex on exosome biogenesis need further exploration.

Exosomes can be released from almost all of the cells infected with virus, which plays an important role in viral infection (van der Grein et al., 2018). Many viruses utilize the exosome generation pathway and interact with ESCRT complex proteins to enhance the viral replication process (Thery et al., 2002). HIV-1 (Human immunodeficiency virus I) utilizes exosomal machinery for HIV-1 RNA and protein trafficking (Madison and Okeoma, 2015). During Epstein-Barr virus (EBV) replication, the ESCRT machinery regulates the virus maturation (Lee et al., 2012). As previously mentioned, EV71 can resort to exosomes to spread between cells and suppress the innate immune response to enhance viral replication (Fu et al., 2017; Huang et al., 2020). Our current work also explored the role of VPS25-mediated exosome in EV71 replication. Employing siRNA silencing and inhibitor of exosome biogenesis, we found out that VPS25-mediated exosome promoted EV71 infection. Considering the fact that exosomes cloak the EV71 virion in a non-lytical manner to benefit viral transmission (Gu et al., 2020), we speculate the VPS25-mediated exosomes could also be engaged in the EV71 transmission and replication. Hence, it could be concluded that VPS25 regulates exosome biogenesis and possibly promotes the release of exosomal progeny EV71, and eventually facilitates EV71 infection and replication.

Besides exosomes, there are also certain roles of some extracellular vesicles’ populations (especially size > 200 nm) in viral replication. For example, intercellular transfer of membrane proteins from microparticles may have implications for HIV-1 infection (Mack et al., 2000). Microvesicles containing infectious virus were readily observed in cultures of differentiated progenitor cells infected with Coxsackievirus B3 (CVB3) (Robinson et al., 2014). There are interesting and compelling findings regarding the importance of exosomes and other extracellular vesicles during viral infection, which will allow for a better understanding of virulence mechanisms and immune responses, as well as the development of new diagnostics and vaccines (Schorey et al., 2015). Notably, the factors in exosomes and other extracellular vesicles involved in EV71 replication require clarification in future work.

In sum, we illuminated that EV71 promotes the secretion of exosomes through its non-structural protein 3A binding to VPS25, thereby facilitating viral replication, which provides insight into the regulatory mechanism of exosome biogenesis. We believe that the understanding of the relationship between EV71 and exosomes will be beneficial to the exploration of viral pathogenesis and the development of antiviral therapy in EV71-associated diseases.

## Data availability statement

The original contributions presented in this study are included in the article/Supplementary material, further inquiries can be directed to the corresponding authors.

## Author contributions

ZR, YL, JW, and ZL: conceptualization and design. ZR, YL, ZC, JY, and CL: data curation. ZC, JY, and CL: formal analysis. ZR, YL, PP, and QZ: methodology. ZC, JY, CL, PP, and QZ: investigation. PP, QZ, JW, and ZL: validation. JW and ZL: funding acquisition, supervision, and writing – review and editing. ZR, YL, and ZL: writing – original draft. All authors had finally approved the version of the manuscript to be published and agreed to be accountable for all aspects of the work.
