# Supplementary material for: Enterovirus 71 non-structural protein 3A hijacks vacuolar protein sorting 25 to boost exosome biogenesis to facilitate viral replication
Source: Front Microbiol. 2022 Oct 5;13:1024899. doi: 10.3389/fmicb.2022.1024899 (PMC9581156; doi:10.3389/fmicb.2022.1024899)
Supplement: Supplementary file 1 [file Data_Sheet_1.pdf]

Supplemental material

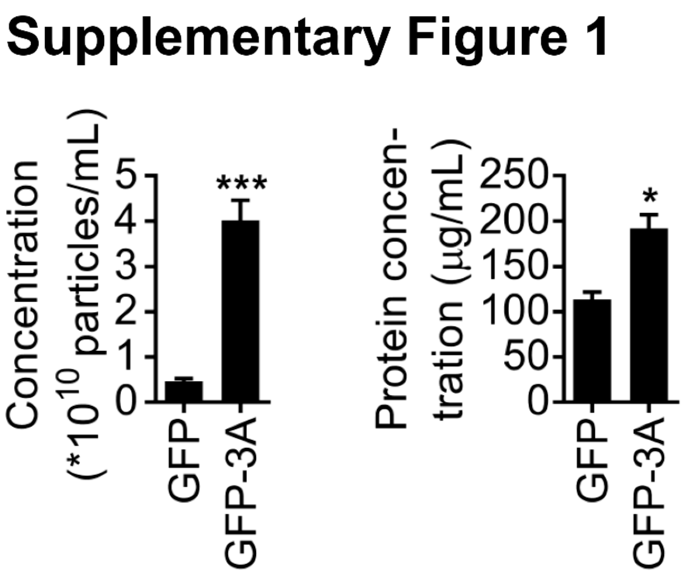

Supplementary Figure 1. The particles and protein concentration of exosomes in the presence of GFP and GFP-3A proteins.

RD cells were transfected with 2 μg plasmids expressing GFP or GFP-tagged 3A protein. At 24 h post-transfection, the extracted exosomes from cell supernatants were measured by size and distribution, respectively. Graph shows as mean ± SEM. *, $P < 0.05$; **, $P < 0.01$; ***, $P<0.001$.

Supplementary Figure 2

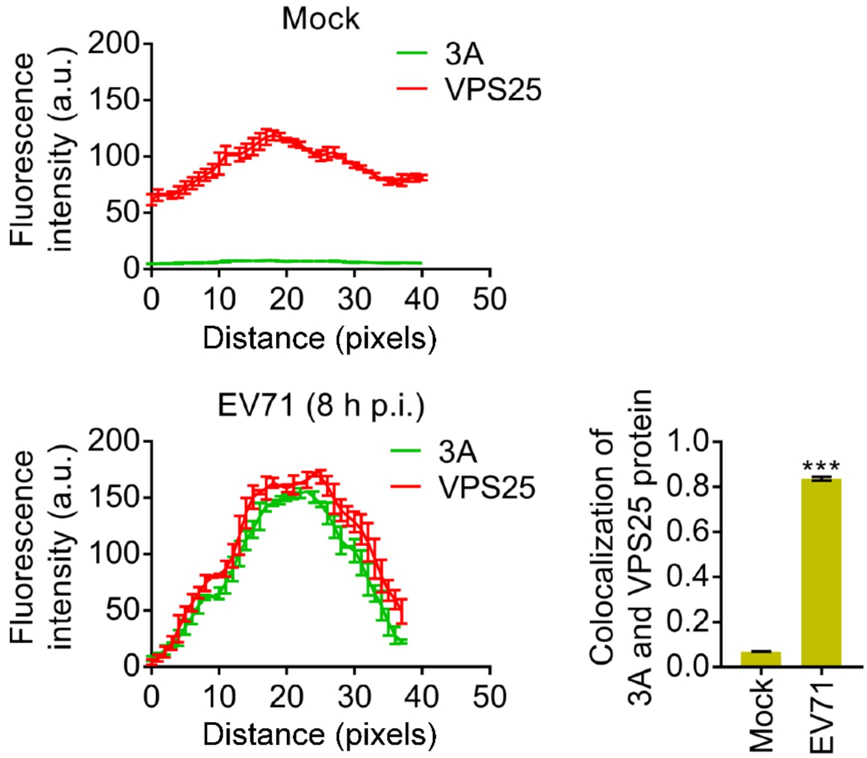

Supplementary Figure 2. Colocalization of VPS25 and EV71 3A proteins.

RD cells were infected with EV71 (MOI = 0.5) for 8 h. Immunofluorescence assay was performed after labeling proteins of EV71 3A (green), VPS25 (red), and staining nuclei with DAPI (blue). The fluorescence intensity of mock and EV71 groups was analyzed using ImageJ software. a.u., arbitrary unit. Graph shows as mean ± SD. *, $P < 0.05$; **, $P < 0.01$; ***, $P<0.001$.
